# Effects of indirubin and isatin on cell viability, mutagenicity, genotoxicity and BAX/ERCC1 gene expression

**DOI:** 10.1080/13880209.2017.1354387

**Published:** 2017-07-25

**Authors:** Manoela Viar Fogaça, Priscila de Matos Cândido-Bacani, Lucas Milanez Benicio, Lara Martinelli Zapata, Priscilla de Freitas Cardoso, Marcelo Tempesta de Oliveira, Tamara Regina Calvo, Eliana Aparecida Varanda, Wagner Vilegas, Ilce Mara de Syllos Cólus

**Affiliations:** a Department of General Biology, Center of Biological Sciences, State University of Londrina, Londrina, Brazil;; b Araraquara Institute of Chemistry, São Paulo State University, Araraquara, Brazil;; c Araraquara Faculty of Pharmaceutical Sciences, Department of Biological Sciences, São Paulo State University, Araraquara, Brazil;; d Experimental Campus of the Paulista Coast, São Paulo State University, São Vicente, Brazil

**Keywords:** *Indigofera suffruticosa*, *Indigofera truxillensis*, HeLa cells, CHO-K1 cells, cytokinesis-blocked micronucleus assay, comet assay, apoptosis

## Abstract

**Context:** *Indigofera suffruticosa* Miller (Fabaceae) and *I. truxillensis* Kunth produce compounds, such as isatin (ISA) and indirubin (IRN), which possess antitumour properties. Their effects in mammalian cells are still not very well understood.

**Objective:** We evaluated the activities of ISA and/or IRN on cell viability and apoptosis *in vitro*, their genotoxic potentials *in vitro* and *in vivo*, and the IRN- and ISA-induced expression of *ERCC1* or *BAX* genes.

**Materials and methods:** HeLa and/or CHO-K1 cell lines were tested (3 or 24 h) in the MTT, Trypan blue exclusion, acridine orange/ethidium bromide, cytokinesis-blocked micronucleus (CBMN) and comet (36, 24 and 72 h) tests after treatment with IRN (0.1 to 200 μM) or ISA (0.5 to 50 μM). Gene expression was measured by RT-qPCR in HeLa cells. Swiss albino mice received IRN (3, 4 or 24 h) by gavage (50, 100 and 150 mg/kg determined from the LD_50_ – 1 g/kg b.w.) and submitted to comet assay *in vivo*.

**Results:** IRN reduced the viability of CHO-K1 (24 h; 5 to 200 μM) and HeLa cells (10 to 200 μM), and was antiproliferative in the CBMN test (CHO-K1: 0.5 to 10 μM; HeLa: 5 and 10 μM). The drug did not induce apoptosis, micronucleus neither altered gene expression. IRN and ISA were genotoxic for HeLa cells (3 and 24 h) at all doses tested. IRN (100 and 150 mg/kg) also induced genotoxicity *in vivo* (4 h).

**Conclusion:** IRN and ISA have properties that make them candidates as chemotherapeutics for further pharmacological investigations.

## Introduction

Naturally occurring compounds, including those extracted from medicinal plants, continue to play an essential role in primary health care, especially in developing countries, and are a promising source of novel bioactive compounds for drug development (Mothana et al. 2009; Cragg and Newman 2013). Specifically, the genus *Indigofera* (Fabaceae) is known to contain several biologically active compounds, such as flavonoid glycosides (Hasan et al. 1994; Calvo et al. 2011), nitro compounds (Garcez et al. 2003) and alkaloids (Chanayath et al. 2002; Calvo et al. 2011). Among *Indigofera* species, *Indigofera suffruticosa* Miller and *Indigofera truxillensis* Kunth, from which the compounds indoxyl and isatin can be isolated, are common plants found in Brazilian savannah (Calvo et al. 2011). Following dimerization, these substances are converted, respectively, into indigo and its isomer, indirubin (Cooksey 2001).

Since the 1980s, when researchers started testing indirubin in clinical trials for the treatment of chronic myelocytic leukaemia, studies have been conducted to evaluate the antitumour properties of this compound and its analogues, named indigoids (Blažević et al. 2015). Nowadays, several pieces of evidence suggest that these drugs present antiproliferative and proapoptotic activities against different types of tumour cell lines (Nam et al. 2005; Perabo et al. 2011; Singh et al. 2012; Cândido-Bacani et al. 2013; Song et al. 2013; Ichimaru et al. 2015).

Indigoids’ mechanisms of action are still under investigation, but several studies suggest that indirubins act as inhibitors of cyclin-dependent kinases (CDKS) and glycogen synthase kinase 3β (GSK3β) in tumour cells, resulting in an impairment of cell cycle progression. Also, these drugs induce apoptosis by inactivation of Stat3, a transcription factor that controls cell proliferation and survival (Polychronopoulos et al. 2004; Nam et al. 2005; Yu et al. 2016). Moreover, indirubin and its derivatives may induce antiproliferative effects through the regulation of growth factors pathways, interfering with the activity of protein kinase B (Akt), extracellular signal-regulated kinases (Erk), Notch1 and cytokines (Sethi et al. 2006; Zhen et al. 2007; Lee et al. 2008; Kim et al. 2011). In the same direction, isatin inhibits cell proliferation and induces apoptosis in mouse and human neuroblastoma cells by altering Erk signaling (Hou et al. 2008). Also, it is suggested that these drugs inhibit protooncogenes, such as *c-myb* (Liu et al. 1996), and activates Bax, a proapoptotic Bcl-2 family member (Shi and Shen 2008).

While there are some studies in the literature unveiling mechanisms that may mediate indigoids’ antitumour activities, so far the genotoxic and mutagenic potentials of indirubin in tumour cells remain poorly investigated. Also, for future clinical proposes, it is very important to assess possible toxic effects of the drug in non-tumour cell lines and *in vivo*. In a previous study, we have demonstrated that isatin did not show a significant mutagenic effect on either Chinese hamster ovary cells (CHO-K1) or human cervical cancer cells (HeLa). However, the drug did reduce cell proliferation and promoted apoptosis in both cell lines (Cândido-Bacani et al. 2013). In addition to these *in vitro* results, isatin was genotoxic and mutagenic in mice bone marrow and peripheral blood cells after 14 consecutive days of treatment, but not after acute injection (Cândido-Bacani et al. 2011).

Similarly, to better clarify some pharmacological effects and safety of indirubin, the present study aimed to verify whether acute treatment could induce cytotoxicity, mutagenicity and genotoxicity in cultured mammalian cells (CHO-K1 and HeLa cells) and in peripheral blood cells. Furthermore, to complement the studies previously performed using acute isatin treatment (Cândido-Bacani et al. 2011, 2013), we evaluated its genotoxic activity *in vitro* and its capacity to reduce cell viability in HeLa cells. Finally, we investigated indirubin- and isatin-induced expression of two genes critical for DNA repair and apoptosis, the enzyme excision repair cross-complementation group 1 (*ERCC1*) and Bcl-2 associated X (*BAX*), respectively, in HeLa cells.

## Materials and methods

### Chemicals

Aerial parts of plants were collected in 2006 in Rubião Junior, Botucatu City, São Paulo State, Brazil, and authenticated by Prof. Dr Jorge Yoshio Tamashiro. The voucher specimens of *I. suffruticosa* (HUEC 129598) and *I. truxillensis* (HUEC 131827) were deposited at the Herbarium of the State University of Campinas (Unicamp), Campinas, São Paulo, Brazil. The compounds were purified at the Institute of Organic Chemistry, UNESP, Campus of Araraquara, Brazil. Initially, indirubin was obtained from aerial parts (1.5 kg) of *I. suffruticosa* (5.0 mg) and *I. truxillensis* (8.0 mg). However, due to the low yield of indirubin isolated from *Indigofera* species (Calvo et al. 2011), it was synthesized in the laboratory to obtain enough compound for the bioassays. The indirubin was produced based on a modified methodology of Ferandin et al. (2006), where isatin reacted with 3-acetoxyindole in alkaline medium to give, in good yields, the bisindole indirubin selectively in the *Z* form.

General procedure for the preparation of indirubin is as follows: isatin (0.91 mmol) was dissolved in methanol (20 mL) and 3-acetoxyindole (0.61 mmol) was added, followed by Na_2_CO_3_ (155 mg). The mixture was stirred under an inert atmosphere (N_2_) for 4 h. The dark product obtained was washed with MeOH/H_2_O (1:1, v/v, 20.0 mL) and filtered. Drying overnight gave the corresponding *Z*-indirubin (126 mg, 80%). Its structure was identified on the basis of the mass spectra. ^1^H and ^13 ^C NMR data were compared to those reported in the literature (Guengerich et al. 2004). Since the yield of isatin obtained from medicinal plants is very low, it was commercially acquired (P.A. ≥ 99%, Fluka, St. Louis, MO, CAS: 91-56-5).

Both compounds were diluted in phosphate buffer solution (PBS, 0.01 M) and used at a proportion of 1% of the total culture medium volume. The doses ranged from 0.1 to 200 μM for indirubin and from 0.5 to 50 μM for isatin. The last doses were defined as non-cytotoxic by the MTT assay (Cândido-Bacani et al. 2013). For the *in vivo* experiments, indirubin was diluted in dimethyl sulphoxide (DMSO, CAS: 67-68-5, Mallinckrodt, Phillipsburg, NJ) to 50% and PBS. The doses of indirubin were determined by the LD_50_ of indirubin (1 g/kg b.w.) using the acute oral toxicity test, according to the Organization for Economic Co-operation and Development (OECD) protocols (OECD 2001). The doses selected for the genotoxicity and mutagenicity tests (50, 100, and 150 mg/kg b.w.) correspond to 5, 10 and 15% of the estimated LD_50_. Cytochalasin B (C29H37NO5 – CAS: 14930-96-2, Sigma) was diluted in dimethyl sulphoxide (DMSO, CAS: 67-68-5, Mallinckrodt) to obtain a stock solution of 2 mg/mL, which was kept at 4 °C in the dark. From this stock solution, we prepared a working solution diluted in PBS (0.3 mg/mL) that was stored at 4 °C in the dark. Cytochalasin B was used in culture medium at a final concentration of 3 μg/mL.

Doxorubicin [DXR, 0.3 μM (Pharmacia & Upjohn, Milan, Italy)] and cyclophosphamide [CPA, 40 mg/kg b.w., (CAS: 50-18-0, Sigma, St. Louis, MO)] were used as positive controls in the *in vitro* and *in vivo* (intraperitoneally) experiments, respectively. PBS was used as a negative control.

### *In vitro* experiments

#### Cell culture

CHO-K1 and HeLa cells were maintained in disposable culture flasks (25 cm^2^ of area, Nunc) at 37 °C in a BOD incubator (Fanem, Bangalore, Karnataka, India). Cells were maintained in complete culture medium (HAM – F10, Sigma + Dulbecco’s modified Eagle’s medium (DMEM), Gibco, Grand Island, NY, 1:1). The medium was supplemented with 10% of foetal calf serum (Gibco), sodium bicarbonate (Reagen, Paraná, Brazil, 1.20 g/L), antibiotics (penicillin 0.06 g/L and streptomycin 0.12 g/L, Sigma) and HEPES (2.38 g/L, Sigma-Aldrich, St. Louis, MO). CHO-K1 and HeLa cells were used between the 3rd and 8th culture passage after thawing. Under these conditions, the cell cycle was approximately 12 h for CHO-K1 cells and 20 h for HeLa cells.

#### Viability assay: MTT (3-[4,5-dimethylthiazol-2-yl]-2,5-diphenyl tetrazolium bromide)

The MTT assay is commonly used to measure cell proliferation and survival. Metabolically active cells cleave the MTT solution within their mitochondria and produce a purple crystal product called formazan (Vellonen et al. 2004). This assay was performed to assess cell viability as described by Mosmann (1983), with some modifications. The concentrations of indirubin used were 0.1 to 200.0 μM. Cells were seeded independently in a 96-well plate with the final volume of 200 μL of complete culture medium containing 2 × 10^4^ cells per well (CHO-K1) or 1 × 10^4^ cells per well (HeLa). The medium was aspirated and cells were exposed to 10 concentrations of indirubin (7 wells for each concentration) and incubated for 3 or 24 h at 37 °C (CHO-K1) or 24 h at 37 °C (HeLa). After treatment, 100 μL of MTT solution (5 mg*/*mL) was added to each well and incubated at 37 °C for 4 h. After incubation, the MTT-containing medium was removed and 200 μL of DMSO was added to each well to dissolve the formazan crystals formed. The absorbances were measured using spectrophotometer (Uniscience) at 550 nm and the results were expressed as cell growth inhibition according to the following formula:

Cell viability (%)=(Absorbance treatment/Absorbance negative-control)×100

where the negative control group was considered as 100% of viable cells.

### Trypan blue exclusion test of cell viability

The Trypan blue exclusion test is a common technique used to determine the number of viable cells in a cell suspension. Unviable cells have membrane damaged and thus stain blue due to the incorporation of the Trypan blue dye. In this test, HeLa cells were treated with indirubin (0.2 to 5.0 μM) or isatin (0.5 to 50 μM) for 3 and 24 h. Cells were then centrifuged (5 min, 1000 rpm) and the pellet was resuspended with culture medium and the Trypan blue dye (0.04%, Acrós Organics, Geel, Belgium). Immediately after, 10 μL was put in a haemocytometer with a coverslip and observed in an inverted optical microscope (Olympus). A total of 200 cells were counted per treatment and the results were expressed as a percentage of viable cells.

### Cytokinesis-blocked micronucleus (CBMN) assay

To assess the mutagenic potential of indirubin, the CBMN test was used to observe the presence of micronucleus in binucleated cells as previously described by Fenech and Morley (1985), with some modifications. Three independent experiments were performed testing five concentrations of indirubin (0.1 to 10.0 μM). These concentrations were deemed appropriate by the MTT test. CHO-K1 cells were treated for 3 and 24 h (Aardema et al. 2006), and HeLa cells were treated for 24 h. After treatment, cultures were exposed to cytochalasin B for 20 h (cells CHO-K1) and 24 h (cells HeLa) to obtain binucleated cells. CHO-K1 cells were resuspended in chilled hypotonic solution of sodium citrate (1%) together with one drop of 1% formaldehyde. Cells were fixed with a 3:1 methanol/acetic acid solution. HeLa cells were resuspended in chilled hypotonic solution of potassium chloride (0.4%), together with one drop of 1% formaldehyde and fixed with a 4:1 methanol/acetic acid solution. Following fixation, the slides were prepared and dyed with Giemsa solution (3%) diluted in phosphate buffer (Na_2_HPO_4_ 0.06 M and KH_2_PO_4_ 0.06 M, pH =6.48) for 5 min, washed with water, dried and kept at 4 °C until microscopic analysis. Cells were analyzed using a microscope (Nikon, Brazil) with a magnification of 40×. The three parameters considered in the cytological analysis were as follows: (i) frequency of micronucleated binucleated cells in 3000 binucleated cells as based on established criteria (Titenko-Holland et al. 1997; Fenech 2000; Lorge et al. 2006), (ii) induction factor (IF) and (iii) nuclear division index (NDI) calculated as previously described by Eastmond and Tucker (1989) using the formula: NDI = [(1 × M1) + (2 × M2) + (3 × M3) + (4 × M4)]/*N*, where M1, M2, M3 and M4 indicate the number of cells with one, two, three and four nuclei, respectively, and *N* is the total number of viable cells. To determine NDI, 500 cells were analyzed by repetition.

### Comet assay *in vitro*

The comet assay was performed using a modified methodology of the alkaline version as proposed by Singh et al. (1988). Cells were treated with indirubin (0.2 to 5.0 μM) or isatin (0.5 to 50 μM) for 3 and 24 h. Next, cells were centrifuged (1000 rpm, 5 min) and 20 μL of the cell suspension was added to 120 μL of low-melting point agarose (Invitrogen). This solution was spread in slides previously covered with normal melting point agarose (Invitrogen). Slides were maintained at 4 °C for 12 h in a membrane lysis solution (2.5 M NaCl, 100 mM EDTA, 10 mM Tris, 10% DMSO, 1% Triton-X, pH =10) and afterwards submitted to an horizontal electrophoresis (300 mA, 25 V, 20 min, 4 °C in the absence of light). Next, slides were neutralized with Tris 0.4 M (pH =7.5), fixed with absolute ethanol and stored at 4° until later analysis. Cells were stained using Gel Red (Uniscience, Miami, EUA) and analyzed using a fluorescence microscope (Nikon, Brazil) with a magnification of 40×, excitation filter of 515–560 nm and emission filter of 590 nm. A total of 100 cells were analyzed by slide. Visual criteria used to evaluate the size of the comets tail were applied as described by Kobayashi et al. (1995). Each treatment’s score was calculated using the following formula (Manoharan and Banerjee 1985):

Score=n(0)×0 +n(1)×1 +n(2)×2 +n(3)×3/N,

where *n*(x) is the number of nucleoids of the x class and *N* is the total number of nucleoids evaluated.

### Apoptosis test

A fluorescent mixture of acridine orange and ethidium bromide (AO/EB) was used to assess apoptosis (McGahon et al. 1995). To do this, 25 μL of the cell suspension obtained in the MN assay, prior to addition of the hypotonic solution, was added to 10 μL of the AO/EB dye (100 μg/mL). Two hundred cells were counted on each slide and classified as follows: (i) normal cells, (ii) apoptotic cells with the presence of apoptotic corpuscles and no change in the membrane (initial apoptosis), (iii) late apoptotic cells with the presence of apoptotic corpuscles and an altered membrane and (iv) necrotic cells. Slides were examined using a Nikon microscope (Brazil) 60 × magnification with excitation filter of 515–560 nm and emission filter of 590 nm. The results are shown as the mean percentage of apoptosis according to the following formula:

% Apoptosis Index (%)= IA + LA/Total×100,

where IA = the number of cells in initial apoptosis and LA = the number of cells in late apoptosis.

### Gene expression

Approximately 1 × 10^6^ HeLa cells were pre-incubated in each experimental culture flask for 24 h. Subsequently, the cells were exposed to 5 μM of indirubin and 50 μM of isatin; PBS was used as a control. Total RNA was extracted using Trizol® reagent (Invitrogen, Life Technologies, Carlsbad, CA) and purified with RNA Miniprep Super Kit (Bio Basic Inc., Markham, Canada) according to the manufacturer’s instructions. RNA integrity was verified using 1% agarose gel electrophoresis. RNA quantification and purity were determined by spectrophotometry (Biophotometer – Eppendorf, Hamburg, Germany). Experiments were performed in duplicate.

cDNA synthesis was carried out in 20 μL reactions of containing 1 μg of total RNA, 40 pmol of oligo dT^12–18^ primer, 10 mM of dNTP Mix, 2 U of RNase out and 10 U of reverse transcriptase M-MLV (Invitrogen, Life Technologies).

Real-time PCR was performed in a PTC 200 DNA Engine Cycler (MJ Research, St. Bruno, Quebec, Canada) using the Chromo 4 detection system (Bio-Rad, Hercules, CA). The reactions were performed in triplicate in a final volume of 20 μL containing 10 μL of Platinum^®^ SYBR^®^ Green qPCR Supermix-UDG (Invitrogen, Life Technologies), 5 pmol of each primer (forward and reverse), and 2 μL of cDNA template. The PCR thermal cycling conditions included an initial step at 50 °C for 2 min and 96 °C for 5 min; 40 cycles at 94 °C for 20 s, 58 °C for 20 s and 72 °C for 20 s; and 72 °C for 2 min. The melting curve analysis was performed at the final stage of the reaction with temperatures ranging from 50 °C and 98 °C every 0.5 °C for 2 s.

The oligonucleotides were designed using the software Gene Runner version 3.05. The sequences are: forward 5′-GATGATTGCCGCCGTGGAC-3′ and reverse 5′-GCCTTGAGCACCAGTTTG-3′ for *BAX* gene; forward 5′-CGAGGAAGCTGGGCGGTA-3′ and reverse 5′-CAAATGTGGTCAGGAGGGTC-3′ for *ERCC1* gene; and forward 5′-GGGCATCCTGGGCTACACT-3′ and reverse 5′-GGTCCAGGGGTCTTACTC-3′ for *GAPDH* gene.

### *In vivo* experiments

#### Animals

Male Swiss albino mice (30 g, 7–8 weeks old) from the Central Animal Farm of the State University of Londrina (UEL) were single-housed (41 × 33 × 17 cm) in a temperature-controlled room (24 ± 2 °C) with a 12 × 12-h light-dark cycle. Female Swiss albino mice were maintained in the same conditions, but in groups of four per cage. Animals received water and food *ad libitum* throughout the study period. All experimental procedures were in accordance with the Ethical Principles in Animal Research adopted by the Brazilian College of Animal Experimentation and were approved by the Ethics Committee on Animal Use of State University of Londrina (CEUA/UEL).

#### Experimental design

To investigate the genotoxic and mutagenic effects of acute indirubin injections, mice were divided into groups of eight, each containing four males and four females for each treatment. Mice were treated with one of three different doses of indirubin: 50, 100 or 150 mg/kg b.w., by gavage [(0.1 mL/10 g of body weight (b.w.)]. After treatment time, animals were anesthetized and euthanized with 2% xylazine HCl combined with 10% ketamine HCl (1:1). All mice were weighted at the beginning and end of each treatment.

#### Micronucleus test of peripheral blood cells

The MN test of mice peripheral blood cells after acute exposure to the drug was used as described by Hayashi et al. (1990). Before treatment with indirubin [50, 100 and 150 mg/kg (time T_0_)] and after 36 h (T_1_), 48 h (T_2_) and 72 h (T_3_), mice blood samples were collected from the tail vein and dropped directly onto slides previously prepared with the dye acridine orange and then covered with cover slips. For each slide, 1000 reticulocytes were counted per animal with the 100 × objective (immersion) and the frequencies of micronucleated cells (MNRETs) were measured using a fluorescence microscope (Nikon, Brazil), combining blue light (488 nm) and yellow filter (515 nm).

#### Comet assay *in vivo*

The *in vivo* comet assay was performed using the alkaline version according to Singh et al. (1988) and Tice et al. (2000) with modifications introduced by da Silva et al. (2000). After 4 or 24 h of treatment with indirubin (50, 100 and 150 mg/kg), heparinized blood samples were collected from the tail vein of mice. Leukocytes were mixed with 0.5% low-melting-temperature agarose (120 μg) in phosphate-buffered saline (PBS) and applied to microscope slides pre-coated with 1.5% agarose in PBS. All subsequent steps were performed as described before (comet assay *in vitro*). After the procedure, slides were stained with ethidium bromide (20 μg/mL). One hundred cells per animal (two slides, 50 cells each) were analyzed under a fluorescence microscope (Nikon, Brazil) with a blue (488 nm) excitation filter and yellow (515 nm) emission (barrier). The visual criterion to evaluate the size of the comet tail was applied as described by Kobayashi et al. (1995), using the formula previously described (comet assay *in vitro*).

#### Statistical analysis

The mean frequency of micronuclei and NDI, scores of DNA damage and the mean percentage of apoptosis were calculated from three independent experiments. Statistically significant differences between control groups and treatments were determined in all experiments using the analysis of variance (ANOVA) test followed by the Tukey test at 95% of confidence. The mean of absorbance obtained in the MTT assay and the mean of viable cells were analyzed using the same statistical tests. All calculations were performed using the SPSS statistics program. The relative expressions of *BAX* and *ERCC1* genes were calculated as described by Pfaffl (2001) using the relative expression software tool (REST) developed by Pfaffl et al. (2002). Data were normalized using the housekeeping gene glyceraldehyde 3-phosphate dehydrogenase (*GAPDH*).

## Results

### Cell viability tests

**Figure 1.**
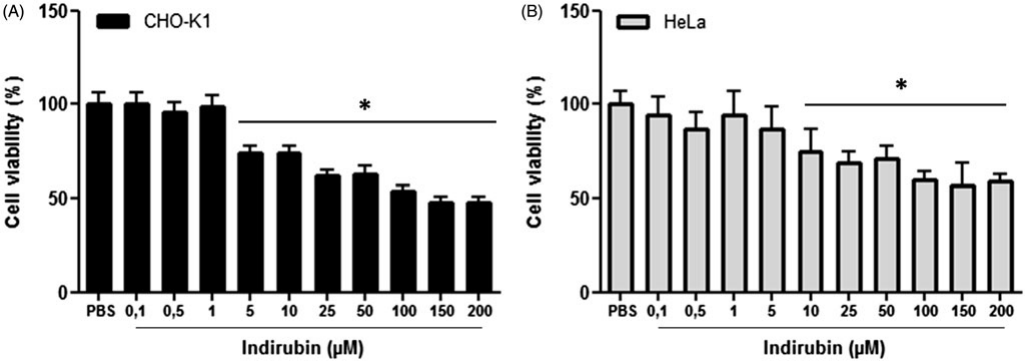
Percentage of CHO-K1 (A) and HeLa (B) cell viability obtained in the MTT test after 24 h of treatment with indirubin. The asterisk indicates significant difference compared to the negative control group (*p* < 0.05, ANOVA followed by Tukey’s test). Each bar represents the mean ± standard deviation of the mean (X ± SD).

**Figure 2.**
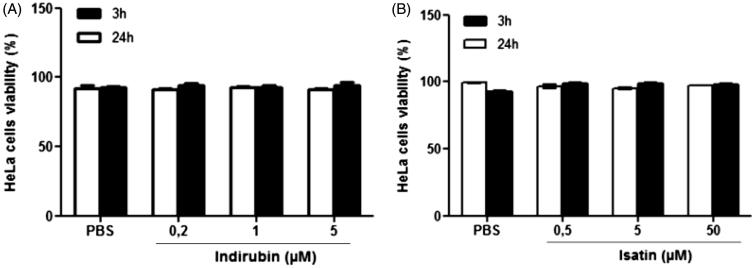
Percentage of HeLa cell viability obtained in the Trypan blue exclusion test after 3 and 24 h of treatment with indirubin (A) and isatin (B). The asterisk indicates significant difference compared to the negative control group (*p* < 0.05, ANOVA followed by Tukey’s test). Each bar represents the mean ± standard deviation of the mean (X ± SD).

In the MTT test, a reduction in CHO-K1 cell viability was observed following a 24-h treatment at doses of 5 to 200 μM of indirubin (*F*_11,72_ = 17.34, *p* < 0.05, Tukey’s test) and in HeLa cells treated at 10 to 200 μM [Figure 1 (*F*_1__1,70_ = 51.85, *p* < 0.05, Tukey’s test)] in comparison with the negative control group. In the Trypan blue exclusion test of cell viability, neither indirubin (0.2, 1 and 5 μM, 3 h: *F*_4,10_ = 12.79; 24 h: *F*_4,10_ = 9.21, *p* > 0.05, Tukey’s test) nor isatin (0.5, 5 and 50 μM, 3 h: *F*_4,10_ = 8.10; 24 h: *F*_4,10_ = 43.37, *p* > 0.05, Tukey’s test) reduced the percentage of HeLa cell viability after 3 or 24 h of treatment (Figure 2).

### Detection of apoptosis in cell culture

The percentage of apoptotic CHO-K1 and HeLa cells observed following treatment with indirubin (0.1 to 10.0 μM) were statistically equal to the negative control group (*p* > 0.05, Tukey’s test) and different from the positive control group after 24 h of treatment (CHO-K1: *F*_6,14_ = 21.04; HeLa: *F*_6,14_ = 6.94, *p* < 0.05, Tukey’s test, Table 1).

**Table 1.** Mean apoptosis index obtained for CHO-K1 and HeLa cells after 24 h of treatment with indirubin.

	Apoptosis Index (%) Mean ± SD	
Treatments	CHO-K1	HeLa
PBS	9.70 ± 0.02	6.17 ± 1.15
DXR 0.3 μM	32.30 ± 0.07*	12.17 ± 1.44*
IRN 0.1 μM	14.75 ± 0.03	7.50 ± 1.41
IRN 0.5 μM	11.17 ± 0.01	7.50 ± 1.41
IRN 1.0 μM	9.90 ± 0.02	7.33 ± 0.29
IRN 5.0 μM	9.80 ± 0.03	5.67 ± 1.04
IRN 10.0 μM	12.00 ± 0.04	8.17 ± 1.04

SD: standard deviation; PBS: phosphate-buffered saline, negative control; DXR: doxorubicin, positive control; IRN: indirubin.

* Indicates significant difference compared to the negative control group (*p* < 0.05, ANOVA followed by Tukey’s test).

### CBMN test *in vitro*

The frequencies of micronucleated CHO-K1 cells observed following treatment with five concentrations of indirubin (0.1 to 10.0 μM) were statistically equal to the negative control group (*p* > 0.05, Tukey’s test) and different from the positive control group after 3 and 24 h of treatment (3 h: *F*_6,14_ = 588.53; 24 h: *F*_6,11_ = 579.15 *p* < 0.05, Tukey’s test). HeLa micronucleated cells were statistically equal to the negative and positive control groups (*F*_6,14_ = 2.53, *p* > 0.05, Tukey’s test) after 24 h of treatment. Thus, indirubin did not induce mutagenicity in CHO-K1 and HeLa cells at the concentrations tested (Table 2).

**Table 2.** Mean frequency of micronucleated binucleated cells (MNBC) in CHO-K1 and HeLa cells obtained in the MN test after 3 and 24 h of treatment with indirubin.

	CHO-K1				HeLa	
	MNBC (3 h)		MNBC (24 h)		MNBC T (24 h)	
Treatments	Mean ± SD	IF	Mean ± SD	IF	Mean ± SD	IF
PBS	7.33 ± 1.53	–	8.00 ± 1.00	–	17.33 ± 6.43	–
DXR 0.75 μg/mL	171.00 ± 10.15*	23.32	207.00 ± 13.90*	25.88	26.00 ± 1.73	1.50
IRN 0.1 μM	9.67 ± 3.22	1.32	12.00 ± 2.65	1.50	14.00 ± 5.29	0.81
IRN 0.5 μM	11.00 ± 2.65	1.50	6.33 ± 0.58	0.79	28.33 ± 1.53	1.63
IRN 1.0 μM	9.33 ± 0.58	1.27	7.00 ± 1.00	0.88	21.00 ± 3.61	1.21
IRN 5.0 μM	9.33 ± 2.31	1.27	10.00 ± 1.00	1.25	25.33 ± 2.52	1.46
IRN 10.0 μM	11.00 ± 2.00	1.50	9.00 ± 1.00	1.13	23.67 ± 11.06	1.37

SD: standard deviation; PBS: phosphate-buffered saline, negative control; DXR: doxorubicin, positive control; IRN: indirubin; IF: induction factor.

* Indicates significant difference compared to the negative control group (*p* < 0.05, ANOVA followed by Tukey’s test).

### Nuclear division index (NDI)

The NDI calculated for CHO-K1 cells by the MN test following 3 h of treatment was not statistically significant at any of the concentrations tested (*F*_6,14_ =  2.57, *p* > 0.05, Tukey’s test). However, after a 24-h treatment, indirubin showed antiproliferative activity at concentrations ranging from 0.5 to 10.0 μM (*F*_6,14_ =  33.58, *p* < 0.05, Tukey’s test). Indirubin also showed antiproliferative activity in HeLa cells at 5.0 and 10.0 μM after 24 h of treatment (*F*_6,14_ =  5.65, Tukey’s test, *p* < 0.05, Table 3).

**Table 3.** Nuclear division index (NDI) calculated in the MN test after treatment of CHO-K1 cells (3 and 24 h) and HeLa cells (24 h) with indirubin.

	CHO-K1		HeLa
Treatments	NDI (3 h) Mean ± SD	NDI (24 h) Mean ± SD	NDI (24 h) Mean ± SD
PBS	1.83 ± 0.02	1.62 ± 0.02	1.73 ± 0.00
DXR 0.3 μM	1.83 ± 0.03	1.63 ± 0.03	1.71 ± 0.01
IRN 0.1 μM	1.85 ± 0.03	1.65 ± 0.04	1.68 ± 0.07
IRN 0.5 μM	1.79 ± 0.01	1.54 ± 0.04*	1.63 ± 0.02
IRN 1.0 μM	1.78 ± 0.04	1.50 ± 0.02*	1.61 ± 0.04
IRN 5.0 μM	1.82 ± 0.03	1.47 ± 0.02*	1.57 ± 0.05*
IRN 10.0 μM	1.84 ± 0.04	1.14 ± 0.03*	1.55 ± 0.09*

SD: standard deviation; PBS: phosphate-buffered saline, negative control; DXR: doxorubicin, positive control; IRN: indirubin; NDI: nuclear division index.

* Indicates significant difference compared to the negative control group (*p* < 0.05, ANOVA followed by Tukey’s test).

### CBMN test *in vivo*

No significant differences were observed between results for males and females (*p* > 0.05, Student’s *t*-test, data not shown); consequently, data for both sexes were combined (Table 4). The frequencies of micronucleated reticulocytes in mice peripheral blood observed following treatment with indirubin (50, 100 and 150 mg/kg) were statistically equal to the negative control group (*p* > 0.05, Tukey’s test) and different from the positive control group after 36, 48 and 72 h of treatment (0 h: *F*_5,42_ =  1.01, *p* > 0.05, Tukey’s test; 36 h: *F*_5,42_ =  145.65; 48 h: *F*_5,42_ =  170.39; 72 h: *F*_5,42_ =  67.51, *p* < 0.05, Tukey’s test, Table 4).

**Table 4.** Frequency of micronucleated reticulocytes (MNRETs) in mice peripheral blood observed after 36, 48 and 72 h of treatment with indirubin.

	MNRET (Mean ± SD)			
Treatments (mg/kg b.w.)	T_0_	T_1_	T_2_	T_3_
PBS	2.50 ± 1.07	2.50 ± 1.31	1.75 ± 1.58	1.88 ± 1,13
DMSO	2.50 ± 1.20	22.00 ± 3.78*	28.63 ± 5.15*	10.25 ± 2.19
CPA 40	3.13 ± 1.46	2.13 ± 0.64	2.88 ± 0.84	1.63 ± 1.06*
IRN 50	2.25 ± 1.91	2.88 ± 1.13	1.63 ± 0.74	1.00 ± 0.76
IRN 100	1.63 ± 1.51	1.88 ± 1.56	2.25 ± 2.28	1.75 ± 0.70
IRN 150	2.00 ± 1.31	2.13 ± 1.13	2.00 ± 1.07	1.38 ± 0.92

Mean ± SD: mean ± standard deviation; PBS: phosphate-buffered saline, negative control; DMSO: dimethyl sulphoxide, DMSO control; CPA: cyclophosphamide, positive control; IRN: indirubin; T_0_: before treatment; T_1_: 36 h; T_2_: 48 h; T_3_: 72 h.

* Indicates significant difference compared to the negative control group (*p* < 0.05, ANOVA followed by Tukey’s test).

### Comet assay

The damage scores obtained from the comet assay in HeLa cells after 3 h of treatment with indirubin (0.2, 1 and 5 μM) and isatin (0.5, 5 and 50 μM) were statistically different from the negative control group (indirubin: *F*_4,10_ =  364.40; isatin: *F*_4,10_ =  38.21, *p* < 0.05, Tukey’s test, Tables 5 and 6) and equal to the positive control group (*p* > 0.05, Tukey’s test) at all doses tested. After 24 h of treatment, indirubin did not induce genotoxicity at the dose of 0.2 μM (*p* > 0.05, Tukey’s test, Table 5), but increased the damage scores in relation to the negative control group at the doses of 1 and 5 μM (*F*_4,10_ =  77.29, *p* < 0.05, Tukey’s test). The values obtained by these doses, however, also differed from the positive control group (*p* < 0.05). Isatin induced genotoxicity after 24 h of treatment at all doses tested compared to the negative control group (*F*_4,10_ =  57.62, *p* < 0.05, Tukey’s test, Table 6) and equal to the positive control group only at 50 μM.

**Table 5.** Mean frequency of damaged cells (DC), distribution of damage classes and scores obtained in the comet assay from HeLa cells after 3 and 24 h of treatment with indirubin.

		Damage levels					
Treatments	Time (h)	0	1	2	3	DC (Mean ± SD)	Score (Mean ± SD)
PBS	3	70.33 ± 2.52	19.00 ± 1.00	10.33 ± 2.52	0.33 ± 0.58	0.30 ± 0.03	0.41 ± 0.06
	24	38.66 ± 9.02	28.00 ± 4.00	21.33 ± 4.51	12.00 ± 1.00	0.61 ± 0.09	1.06 ± 0.14
DXR 0.3μM	3	5.33 ± 5.51	12.67 ± 6.51	17.00 ± 7.94	65.33 ± 5.86	0.95 ± 0.06	2.43 ± 0.06*
	24	0.66 ± 1.15	10.33 ± 1.53	11.33 ± 1.53	77.66 ± 2.52	0.99 ± 0.01	2.66 ± 0.06*
IRN 0.2 μM	3	6.67 ± 3.51	61.33 ± 3.51	20.67 ± 2.52	11.33 ± 2.52	0.93 ± 0.04	1.37 ± 0.06*
	24	31.33 ± 7.57	17.33 ± 2.31	18.66 ± 8.14	32.66 ± 10.97	0.68 ± 0.07	1.52 ± 0.22#
IRN 1 μM	3	10.00 ± 1.00	71.00 ± 7.21	14.00 ± 3.46	5.00 ± 4.36	0.90 ± 0.01	1.14 ± 0.10*
	24	9.33 ± 2.52	14.00 ± 2.00	30.00 ± 5.29	46.66 ± 4.93	0.90 ± 0.02	2.14 ± 0.05*#
IRN 5 μM	3	1.67 ± 0.58	47.33 ± 4.04	36.33 ± 8.62	14.67 ± 5.51	0.98 ± 0.01	1.64 ± 0.04*
	24	5.66 ± 2.52	10.00 ± 2.00	23.00 ± 5.57	61.33 ± 5.51	0.94 ± 0.02	2.40 ± 0.07*#

SD: standard deviation; PBS: phosphate-buffered saline, negative control; DXR: doxorubicin, positive control; IRN: indirubin; DC: damaged cells.

* Indicates significant difference compared to the negative control group.

#Indicates significant difference compared to the positive control group (*p* < 0.05, ANOVA followed by Tukey’s test).

**Table 6.** Mean frequency of damaged cells (DC), distribution of damage classes and scores obtained in the comet assay from HeLa cells after 3 and 24 h of treatment with isatin.

		Damage levels					
Treatments	Time (h)	0	1	2	3	DC (Mean ± SD)	Score (Mean ± SD)
PBS	3	97.00 ± 1.00	3.00 ± 1.00	0.00 ± 0.00	0.00 ± 0.00	0.03 ± 0.01	0.03 ± 0.01
	24	78.00 ± 1.00	18.00 ± 1.00	4.00 ± 2.00	0.00 ± 0.00	1.06 ± 0.14	1.06 ± 0.14
DXR 0.3 μM	3	5.33 ± 2.52	4.33 ± 3.51	4.00 ± 2.65	86.33 ± 4.73	2.71 ± 0.08	2.71 ± 0.08*
	24	0.00 ± 0.00	9.33 ± 4.16	17.00 ± 0.00	73.66 ± 4.16	2.60 ± 0.06	2.60 ± 0.06*
ISA 0.5 μM	3	25.00 ± 12.53	8.00 ± 9.54	4.33 ± 3.21	62.67 ± 19.30	2.05 ± 0.53	2.05 ± 0.53*
	24	11.33 ± 3.78	27.00 ± 7.81	24.00 ± 4.36	37.66 ± 7.23	1.52 ± 0.22	1.52 ± 0.22*#
ISA 5 μM	3	16,00 ± 9.54	23.33 ± 11.02	6.33 ± 4.04	61.00 ± 17.05	2.19 ± 0.33	2.19 ± 0.33*
	24	10.33 ± 1.53	14.66 ± 4.16	21.00 ± 1.00	54.00 ± 4.58	2.14 ± 0.05	2.14 ± 0.05*#
ISA 50 μM	3	9.00 ± 2.00	10.67 ± 1.53	12.33 ± 6.81	71.33 ± 12.22	2.49 ± 0.25	2.49 ± 0.25*
	24	0.00 ± 0.00	11.33 ± 3.51	16.33 ± 2.08	72.33 ± 5.51	2.48 ± 0.19	2.48 ± 0.19*

SD: standard deviation; PBS: phosphate-buffered saline, negative control; DXR: doxorubicin, positive control; ISA: isatin; DC: damaged cells.

* Indicates significant difference compared to the negative control group.

#Indicates significant difference compared to the positive control group (*p* < 0.05, ANOVA followed by Tukey’s test).

In the *in vivo* comet assay, indirubin induced genotoxicity in peripheral blood cells at the higher doses (100 and 150 mg/kg) after 4 h of treatment compared to both the negative and positive control groups (*p* < 0.05, Tukey’s test). However, after 24 h, indirubin failed to induce genotoxicity at all doses tested (*p* > 0.05, Tukey’s test). No significant differences were observed between males and females (Student’s *t*-test, *p* > 0.05, data not shown); consequently, data for both sexes were combined (Table 7).

**Table 7.** Mean frequency of damaged cells (DC), distribution of damage classes and scores obtained in the comet assay from the peripheral blood after 4 and 24 h of treatment with indirubin.

		Damage levels					
Treatments (mg/kg b.w.)	Time (h)	0	1	2	3	DC (Mean ± SD)	Score (Mean ± SD)
PBS	4	90.25 ± 1.58	9.50 ± 1.31	0.13 ± 0.35	0.13 ± 0.35	9.63 ± 1.41	0.10 ± 0.02
	24	87.14 ± 3.24	11.71 ± 1.89	0.86 ± 1.07	0.29 ± 0.76	12.86 ± 3.24	0.15 ± 0.06
CPA 40	4	61.86 ± 6.26	34.71 ± 4.50	2.14 ± 1.57	1.29 ± 0.95	38.14 ± 6.26	0.42 ± 0.09*
	24	61.17 ± 8.52	34.83 ± 7.57	3.33 ± 2.25	0.67 ± 0.82	38.83 ± 8.52	0.44 ± 0.10*
DMSO	4	90.63 ± 1.92	8.88 ± 1.46	0.50 ± 0.93	0.00 ± 0.00	9.36 ± 1.92	0.10 ± 0.03
	24	85.75 ± 2.92	13.13 ± 2.17	1.13 ± 0.99	0.00 ± 0.00	14.25 ± 2.92	0.15 ± 0.04
IRN 50	4	90.14 ± 1.95	9.00 ± 1.29	0.71 ± 1.11	0.14 ± 0.38	9.86 ± 1.95	0.11 ± 0.03
	24	87.29 ± 4.31	12.14 ± 4.18	0.63 ± 1.06	0.25 ± 0.71	12.71 ± 4.31	0.13 ± 0.04
IRN 100	4	80.43 ± 4.69	17.14 ± 4.71	1.86 ± 1.07	0.57 ± 0.79	19.57 ± 4.69	0.20 ± 0.04*#
	24	86.63 ± 3.16	12.25 ± 3.62	0.63 ± 1.06	0.25 ± 0.71	13.13 ± 3.36	0.14 ± 0.03
IRN 150	4	83.63 ± 4.78	15.75 ± 4.68	0.63 ± 0.92	0.00 ± 0.00	16.38 ± 4.78	0.17 ± 0.05*#
	24	87.13 ± 3.40	11.38 ± 3.42	1.25 ± 1.28	0.25 ± 0.07	12.88 ± 3.40	0.15 ± 0.04

SD: standard deviation; PBS: phosphate-buffered saline, negative control; CPA: cyclophosphamide, positive control; DMSO: dimethyl sulphoxide, DMSO control; IRN: indirubin; DC: damaged cells.

* Indicates significant difference compared to the negative control group.

# Indicates significant difference compared to the positive control group (*p* < 0.05, ANOVA followed by Tukey’s test).

### Gene expression

**Figure 3.**
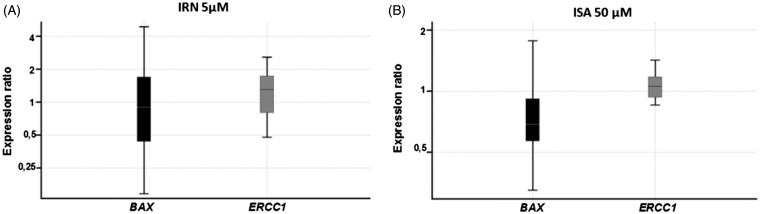
Relative expression of *BAX* and *ERCC1* genes after exposure of HeLa cells to (A) 5 µM of indirubin for 24 h and (B) to 50 µM of isatin for 24 h. Data represent mean values ± SD.

Neither indirubin nor isatin altered *BAX* (one-way ANOVA, *p* = 0.06) or *ERCC1* (*p* = 0.06) gene expression in HeLa cells, respectively, in comparison with controls (Figure 3).

## Discussion

Our data show that indirubin reduced cell viability in the MTT test in both CHO-K1 and HeLa cells without inducing apoptosis. Also, indirubin decreased the nuclear division index in the CBMN assay in both cell lines, indicating antiproliferative activity, and did not exhibit mutagenicity *in vitro* and *in vivo*. However, indirubin and isatin were genotoxic, suggesting that the DNA damage induced by the compounds could be repaired and thus not converted to a permanent mutation. Finally, although not statistically significant, treatment with indirubin and isatin induced a strong trend towards decreasing *BAX* and increasing *ERCC1* gene expression, respectively.

Initially, we performed the MTT assay and the Trypan blue exclusion test to evaluate the capacity of indirubin in reducing cell viability and to define the range of concentrations to be used in the later experiments. In the MTT test, which measures the metabolic activity of the cell by producing colorimetric parameters for analysis (Ferandin et al. 2006), a 24-h treatment with indirubin produced cytotoxic effects in CHO-K1 (5 to 200 μM) and HeLa cells (10 to 200 μM). At lower doses (0.1 to 5 μM), no effect was found in the Trypan blue exclusion test in HeLa cells after treatment with indirubin or isatin. Accordingly, we have previously demonstrated that isatin reduces HeLa cells’ viability in the MTT test at doses higher than 500 μM (Cândido-Bacani et al. 2013). The NDI obtained in the CBMN assay indicated that indirubin induces antiproliferative activity in CHO-K1 cells after 24 h of treatment at concentrations ≥0.5 μM. In HeLa cells, this response was found at the same effective concentration observed in the MTT assay (10 μM), indicating that the decrease in cell viability observed could be due to an arrest in cell division.

The antiproliferative and cytotoxic activity of indirubin and its derivatives was reported in several studies using different cell lines and experimental approaches. Similar to our results, doses higher than 5 μM of indirubin-3′-monoxime, an indirubin analogue, reduced the viability of renal cancer cells after 24-h treatment (Perabo et al. 2006). The drug was also shown to induce cytotoxicity in Hep-2 human laryngeal carcinoma cells (Nam et al. 2005). A 24-h treatment with isatin-Schiff base copper (II) complexes did not alter the viability in the Trypan blue exclusion test in two tumour cell lines: the human neuroblastoma (SH-SY5Y) and promonocytic (U937) cells (Cerchiaro et al. 2005). Also, indirubin inhibited cell proliferation in several cancerous cell lines (Han 1994; Hoessel et al. 1999; Damiens et al. 2001; Marko et al. 2001; Nam et al. 2005; Moon et al. 2006; Perabo et al. 2006; Choi et al. 2010). However, other analogues generated by substitution at various positions of the bisindole skeleton usually require lower concentrations to induce antiproliferative effects, because of its higher solubility, better pharmacological properties and higher potency. Thus, most studies found in literature test synthetic substituted indirubin, such as indirubin-3′-monoxime, indirubin-5-sulphonic acid or 5-chloro-indirubin (Han 1994; Hoessel et al. 1999; Damiens et al. 2001; Marko et al. 2001; Nam et al. 2005; Moon et al. 2006; Perabo et al. 2006; Choi et al. 2010).

Although our results indicated that indirubin reduced cell viability and cell division, apoptosis/necrosis was not observed in HeLa and CHO-K1 cells. Similarly, indirubin-3′-monoxime (>5 μM) did not induce apoptosis in human transitional cell carcinoma (RT4 and T24 cells), although the same concentrations inhibited cell proliferation in a cytotoxicity assay (Perabo et al. 2006). In the present study, the doses of indirubin used to assess induction of apoptosis ranged from 0.1 to 10 μM. Most studies indicate that indirubin and its derivatives prevent cell cycle progression and induce cell death at concentrations above or equal to 10 μM. This effect was observed in SV-40 transformed human breast epithelial cells (HBL-100) (Choi et al. 2010), human transitional cell carcinoma RT112 (Kameswaran and Ramanibai 2009), human breast cancer cells [(MDA-MB-468 and MDA-MB-435 (Moon et al. 2006)], human mammary carcinoma [MCF-7 (Hoessel et al. 1999)] and renal cancer cells (Perabo et al. 2006, 2011). In contrast, indirubin-3′-monoxime caused programmed cell death in Hep-2 cells at lower concentrations (Nam et al. 2005). Differences found in the literature are probably because these activities seem to depend on the cell line used, the duration of treatment and the structure of the compound. On the other hand, a previous study found that 24-h treatment with isatin induced apoptosis at concentrations of 10 and 50 μM (Cândido-Bacani et al. 2013).

The mechanisms by which indigoids exert antiproliferative and cytotoxic effects still needs to be better understood. Several pieces of evidence suggest that indirubin derivatives selectively arrest cell division by inhibiting phosphorylase kinases that regulate the cell cycle, such as CDKs, Akt, Erk and GSK3β (Polychronopoulos et al. 2004; Nam et al. 2005; Lee et al. 2008; Kim et al. 2011; Begum et al. 2015; Yu et al. 2016). Also, these drugs induce apoptosis in tumour cells by blocking protein signal transducer and activator of transcription, particularly Stat3 protein, which is constitutively expressed in most cancerous cells but not in non-tumour cells (Nam et al. 2005; Zhang et al. 2015). Inactivation of Stat3 by indirubin-3′-monoxime (10 μM) and other derivative, E804, resulted in downregulation of anti-apoptotic proteins, such as Mcl-1 and survivin, and subsequent induction of programmed death in Cal-27 and HSC-3 oral cancer cell lines, as well as in human breast and prostate cancer cells (Nam et al. 2005; Lo and Chang 2013). In addition, it was shown that indirubin-3′-monoxime is an inhibitor of death-associated protein kinases (DAPKs) (Jung et al. 2016). Recently, a study using Hoechst 33342 and annexin-V-propidium iodide staining revealed that the indirubin derivative indirubin-3′-epoxide induces caspase-independent apoptosis in human neuroblastoma cells, probably due to a DNA fragmentation and impairment of DNA repair (Kurita et al. 2016). In addition to these mechanisms, indirubins can induce antiproliferative activities by binding the aryl hydrocarbon receptors (AhR), a ligand-activated transcription factor mainly involved in the regulation of xenobiotic-metabolizing enzymes (Adachi et al. 2001; Knockaert et al. 2004).

The magnitude of DNA damage induced by novel therapeutic targets is of great importance in order to determine its toxicity and safety. In the present study, the comet assay was used to evaluate the genotoxic potential of indirubin and isatin. We found that indirubin induced DNA damage *in vitro* and *in vivo*. Isatin was genotoxic *in vitro* at all doses tested after 3 and 24 h of treatment. However, the CBMN assay *in vitro*, using CHO-K1 and HeLa cells, and *in vivo*, in which we evaluated peripheral blood reticulocytes, revealed the absence of mutagenicity for indirubin at all concentrations tested. Thus, these results added to those obtained by Cândido-Bacani et al. 2011, 2013), in which mutagenicity was not detected *in vitro*, indicate that the cell damage detected could be repaired by the DNA repair system and thus not converted to permanent mutations. Thus, the absence of mutagenicity after indirubin treatment using CHO-K1 cells can serve as initial evidence that this compound may be safe for mammalian use, once it did not harm the genome. A recent study demonstrated that an aqueous extract of Perscicariae Rhizoma containing low concentrations of indirubin (0.009%) and indigo (0.043%) did not produce genotoxicity, chromosomal aberrations or micronucleated polychromatic erythrocytes from bone marrow (Lee et al. 2016).

Due to studies exploring the apoptotic activities of indirubin and isatin and considering the possibility that the damage caused by these compounds are repaired, we investigated the expression of two genes involved in these processes, the *ERCC1* and *BAX*. ERCC1 has an important role in nucleotide excision repair (NER) pathway (De Laat et al. 1998). ERCC1 forms a heterodimeric complex with Xeroderma pigmentosum F (XPF), and the ERCC1-XPF complex has a structure-specific endonuclease activity, which cleaves the damaged strand at the 5′ end around the lesion (Sijbers et al. 1996; De Laat et al. 1999). *BAX* gene is important member that actively participates in the apoptotic mechanisms. It has been shown that the mitochondrial Bcl-2 protein family plays an essential role in this mechanism. This family includes anti-apoptotic proteins, e.g., Bcl-2, and proapoptotic proteins, such as Bax (Hou et al. 2008; Song et al. 2013).

Although indirubin and isatin produced a trend in decreasing *BAX* and increasing *ERCC1* gene expression in HeLa cells after 24 h of treatment, respectively, we failed to detect any statistical significance. These results indicate that the absence of mutagenicity and the proapoptotic effect of isatin (Cândido-Bacani et al. 2013) may not be exclusively modulated by the NER pathway. A previous study showed that isatin increased the expression of the *CASP-3* gene. This gene is involved in both intrinsic and extrinsic apoptosis pathways, encoding the protein caspase 3, which is responsible for activating endonucleases that initiate the process of DNA cleavage (Chang and Yang 2000; Grutter 2000; Hengartner 2000).

It has been shown that 48-h treatment with isatin (50, 100, 200 μmol/L) can exert proapoptotic activity in human neuroblastoma cells and mouse neuroblastoma cells (SH-SY5Y) *in vitro* (Song et al. 2013), accompanied by a decrease in the expression of *BCL*-*2* mRNA. Moreover, while it did not modulate the expression of *BAX* mRNA, the *BCL-2*/*BAX* ratio was decreased (Song et al. 2013). Also, indirubin-3′-monoxime induced apoptosis in HeLa cells through the extrinsic pathway by modulating Bid and Bax proteins (Shi and Shen 2008).

However, in the present study indirubin did not induce significant changes in *BAX* gene expression and it was not found a proapoptotic effect in the acridine orange and ethidium bromide staining test. Thus, genes involved in other forms of cell death, as those regulated by death receptors in the extrinsic pathway, must be investigated. Also, as our results indicated that the DNA damage induced by indirubin and isatin *in vitro* and *in vivo* could be effectively repaired, more studies are needed to verify the genes involved in this process, such as the MMR, BER or DSB genes.

## Conclusion

The results found in the present study showed that isatin and indirubin compounds might be candidates for further investigations directed at their use as chemotherapeutic substances, as results obtained in the present work and previous experiments suggest that these drugs display cytotoxic and antiproliferative activities and with a lack of mutagenicity.
